# Glycine Betaine Accumulation, Significance and Interests for Heavy Metal Tolerance in Plants

**DOI:** 10.3390/plants9070896

**Published:** 2020-07-15

**Authors:** Shafaqat Ali, Zohaib Abbas, Mahmoud F. Seleiman, Muhammad Rizwan, İlkay YAVAŞ, Bushra Ahmed Alhammad, Ashwag Shami, Mirza Hasanuzzaman, Dimitris Kalderis

**Affiliations:** 1 Department of Environmental Sciences and Engineering, Government College University, Allama Iqbal Road, Faisalabad 38000, Pakistan; zohaib.abbas83@gmail.com (Z.A.); mrazi1532@yahoo.com (M.R.); 2 Department of Biological Sciences and Technology, China Medical University, Taichung 40402, Taiwan; 3 Plant Production Department, College of Food and Agriculture Sciences, King Saud University, P.O. Box 2460, Riyadh 11451, Saudi Arabia; mseleiman@ksu.edu.sa; 4 Department of Crop Sciences, Faculty of Agriculture, Menoufia University, Shibin El-kom 32514, Egypt; 5 Department of Plant and Animal Production, Kocarli Vocational High School, Aydın Adnan Menderes University, 09100 Aydın, Turkey; iyavas@adu.edu.tr; 6 Biology Department, College of Science and Humanity Studies, Prince Sattam Bin Abdulaziz University, Al Kharj Box 292, Riyadh 11942, Saudi Arabia; b.alhamad@psau.edu.sa; 7 Biology Department, College of Sciences, Princess Nourah bint Abdulrahman University, Riyadh 11617, Saudi Arabia; AYShami@pnu.edu.sa; 8 Department of Agronomy, Faculty of Agriculture, Sher-e-Bangla Agricultural University, Dhaka 1207, Bangladesh; mhzsauag@yahoo.com; 9 Department of Electronics Engineering, Hellenic Mediterranean University, 73100 Chania, Crete, Greece; kalderis@hmu.gr

**Keywords:** heavy metal stress, glycine betaine (GB) accumulation, exogenous application, plants, antioxidant enzymes, genetic engineering

## Abstract

Unexpected biomagnifications and bioaccumulation of heavy metals (HMs) in the surrounding environment has become a predicament for all living organisms together with plants. Excessive release of HMs from industrial discharge and other anthropogenic activities has threatened sustainable agricultural practices and limited the overall profitable yield of different plants species. Heavy metals at toxic levels interact with cellular molecules, leading towards the unnecessary generation of reactive oxygen species (ROS), restricting productivity and growth of the plants. The application of various osmoprotectants is a renowned approach to mitigate the harmful effects of HMs on plants. In this review, the effective role of glycine betaine (GB) in alleviation of HM stress is summarized. Glycine betaine is very important osmoregulator, and its level varies considerably among different plants. Application of GB on plants under HMs stress successfully improves growth, photosynthesis, antioxidant enzymes activities, nutrients uptake, and minimizes excessive heavy metal uptake and oxidative stress. Moreover, GB activates the adjustment of glutathione reductase (GR), ascorbic acid (AsA) and glutathione (GSH) contents in plants under HM stress. Excessive accumulation of GB through the utilization of a genetic engineering approach can successfully enhance tolerance against stress, which is considered an important feature that needs to be investigated in depth.

## 1. Introduction

Over the years metalloids and heavy metals have received substantial consideration in multidisciplinary areas of environmental and geosciences due to their biomagnification, bioaccumulation along with negative ecological impacts [1,2,3,4,5,6,7,8,9,10]. Anthropogenic activities are the main source of HM pollution for both water bodies and soil, as a result causing severe problems for human being as well [11,12,13,14]. Smelting, electroplating, mining, fertilizers, open waste dumping, tanneries, automobile emission, electronic and paper industries have been reported as discharging huge amounts of HMs in the environment as an anthropogenic source [15,16,17]. Certainly, high exposure to heavy metal pollution has harmful effects on human health, including cancer, reproductive and neurological disorders and damage to central nervous system [18]. Human health could be exposed to different HMs in different ways, such as generation of HMs from various sources can produce pollution for the plants consumed by humans [19]. Heavy metals are hazardous pollutants characterized by their toxicity, persistence and non-biodegradability in neighboring environments [20]. Depending upon the concentration of HMs in the surrounding environment and the characteristics of plant species, metals can gather to various degrees in plants. 

Plants have the capability to uptake and translocate toxic metals in different parts of their body as reported in several studies [21,22,23,24,25,26]. Uptake of HMs in plants not only inhibits the growth attributes of plants, but also induces toxic effects on consumer health [27,28,29,30,31,32]. Various studies have revealed the harmful impacts of HMs on human health and plant growth and development [33,34,35,36,37,38,39]. High concentrations of harmful metals particularly in soil significantly affects physiological and morphological traits of the plants [40,41]. Under HM stress, plants showed evident symptoms of structural transformation and inhibition of the photosynthesis process [42]. Different HMs such as zinc (Zn), cadmium (Cd), aluminum (Al), chromium (Cr), nickel (Ni) and metalloids like arsenic (As) diminish plant development and growth by producing a number of metabolic alterations in plants [43,44]. Most likely, heavy metal ions stay in the cytoplasm and cause oxidative stress through the excessive formation of reactive oxygen species (ROS), which ultimately restrict cell metabolism [45].

## 2. Signaling Response in Plants against Heavy Metal Stress 

Environmental stresses in plants are caused by intense growth conditions that restrain the normal development and growth of the plants, which may be fatal in severe cases [46]. Different types of environmental stresses like HMs, temperature, drought and salinity are main cause of morphological and physiological changes in plants. All environmental stresses (biotic and a biotic) generate oxidative stress which can easily harm cell components and cause their dysfunction pursued by the uptake and over production of ROS at high rates. Enzyme complexes of nicotinamide adenine dinucleotide phosphaten (NADPH) oxidases generally involved in the generation of ROS, which then usually accumulate in different organelles of cell especially cytoplasm, mitochondria and nucleus [47,48]. Uncontrolled production of ROS results in protein denaturation, carbohydrates oxidation, oxidation of RNA and DNA, lipid peroxidation in cellular compartments, and it severely affects enzymatic activity in plants [49]. 

**Figure 1:**
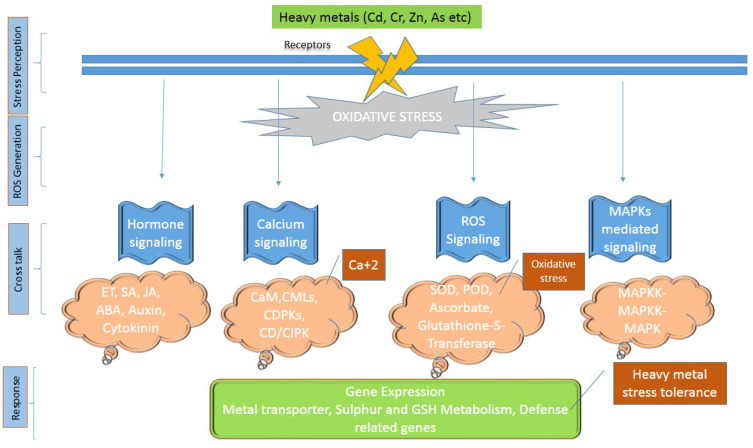
Heavy metals stress signaling in plants.

Heavy metals produce oxidative stress by disturbing the ROS stability in cells, and then induce the antioxidant mechanism of plants. For examples, excessive accumulation of Cr leads towards the generation of ROS and without doubt oxidative stress as well [50,51]. Heavy metals at toxic levels obstructs normal metabolic processes in different ways including displacement and disturbance of protein building blocks [52], adversely altering and affecting the authenticity of the cytoplasmic membrane, repressing critical events in plants such as respiration, photosynthesis and enzymatic activities [53]. In plants, heavy metal stress starts diverse signaling paths including signaling of mitogen activated protein kinase mitogen activated protein kinases (MAPKs) are group of protein kinases that perform a vital role during isignal transduction through modulating gene transcription in the nucleus as an appropriate response to changes occurs in the cellular environment, calcium dependent signaling, hormone signaling and signaling of ROS [54]. These signaling pathways increase the expression of responsive stress genes in plants [55]. Transduction of heavy metal stress signaling is started by ion channels/receptors through awareness of stress signals and also through non-protein messengers like hydrogen ions, calcium and cyclic nucleotides as given in (Figure 1). Several studies reported the importance of these signaling molecules and responsive genes in plants against the stress induced by HMs [56,57,58,59,60].

Heavy metals stress provoke oxidative stress, osmotic stress and denaturation of proteins in plants, which eventually lead adaptive cellular responses such as acceleration of antioxidant system, speeding up the ROS scavenging, initiation of stress protein and accumulation of well-matched solutes [61]. Commonly, plants produce various types of solutes in response of stress. Most common compatible solutes are polyols, proline, sucrose, trehalose, different compounds of quaternary ammonium like choline O-sulfate, pipecolatebetaine, hydroxyprolinebetaine, alanine betaine and glycine betaine (GB). They are also known as osmoprotectants because they protect plant from dehydration injuries. They have the ability to safeguard plants from harmful stress through various ways such as the stabilization of protein and enzymes as well as safeguarding of membrane integrality and detoxification of ROS [62,63,64]. Application of these compatible solutes can significantly improve crop growth and yield in stressed environment.

## 3. Accumulation of Glycine Betaine (GB) as an Emerging Signal Molecule in Plants 

Many plants accumulate higher concentration of GB against abiotic stress [65]. GB mostly is accumulated in chloroplast, where it is actively involved in safeguarding the Photosystem II (PSII) effectiveness under stressful conditions [64,66]. Accumulation and uptake of GB is more prominent in chloroplasts as compared to other cellular compartments of the plants in shielding the plants facing salinity and oxidative stress [67]. According to He et al. [68], high accumulation of GB enhances the rate of seed germination along with exceptional growth of grains., Accumulation of GB in plants is beneficial because it provides the nitrogen source as a result of better roots growth and germination of seeds. The better growth and earlier sprouting of roots results in greater biomass of wheat seedlings [68]. This suggests that more accumulation of GB results in more enhancements of both the seedlings and seedlings growth in wheat crop [68]. Not all the plants have the mechanism to accumulate GB by natural means, even at lower levels some of the transgenic plants have ability to accumulate GB [69].

Glycine betaine triggers the antioxidant system of the plant by activating the activities of diverse antioxidant enzymes like peroxidase (POX), catalase (CAT) and superoxide dismutase (SOD), which ultimately protects plant from oxidative harm [70]. Stimulation of antioxidant machinery in different plants species under numerous stressful condition by GB is extensively studied and reported in different studies [71,72,73]. Moreover, GB not only improves the development and growth of the plant, but also increases yield through employing much better photosynthesis system in plant [74]. Fundamental mechanisms involve in stress tolerance and degree of yield development by GB is yet not fully exposed. Taking into account the importance of GB, present review highlights the effectiveness and efficiency of GB in remediating the stress induced by HMs along with improvement in plant growth and development. 

## 4. Biosynthesis of Glycine Betaine in Plants

**Figure 2:**
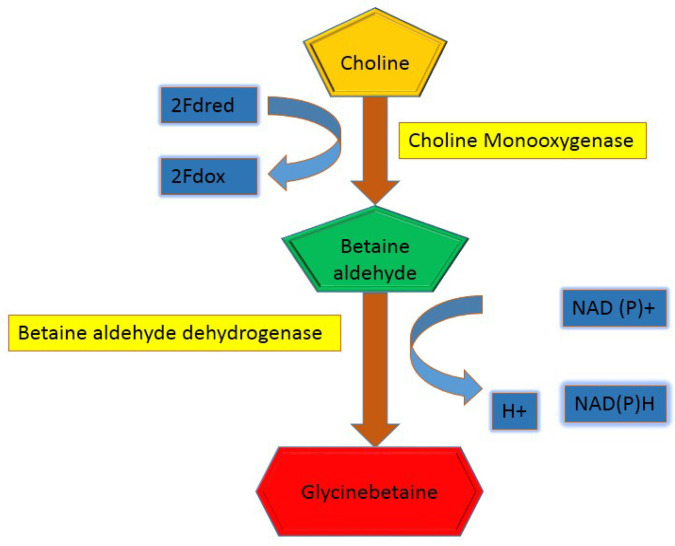
Biosynthesis pathway of glycine betaine in higher plants.

Various kinds of procedures and techniques have been utilized to cut down the heavy metal stress in plants. Use of different osmoprotectants to decrease the stress in plants induced by metal toxicity is one such technique. Glycine betaine (N,N,N-trimethylglycine) is a very vital osmoregulation material, and its concentration differs significantly amongst different species of plants. Glycine betaine in higher plants synthesized in chloroplast from serine through betaine aldehyde, choline and ethanolamine [75]. Choline is altered into betaine aldehyde with the help of choline mono oxygenase, which then eventually converts into glycinebetaine (GB) via betaine aldehyde hydrogenase (Figure 2). Under stressful conditions various plants have the potential to generate the GB as vital compound. Glycine betaine is environmentally safe, non-toxic and water soluble compound [66]. Glycine betaine is extensively engaged in the fortification of plants alongside various stress full conditions including salinity [76,77], drought [78,79,80], and HMs [81,82,83,84,85]. Application of GB reduced the HMs stress on plants by stabilizing the proteins, hunting ROS and protecting photosynthesis process [86].

## 5. Role of Glycine Betaine in Plant Growth

**Figure 3:**
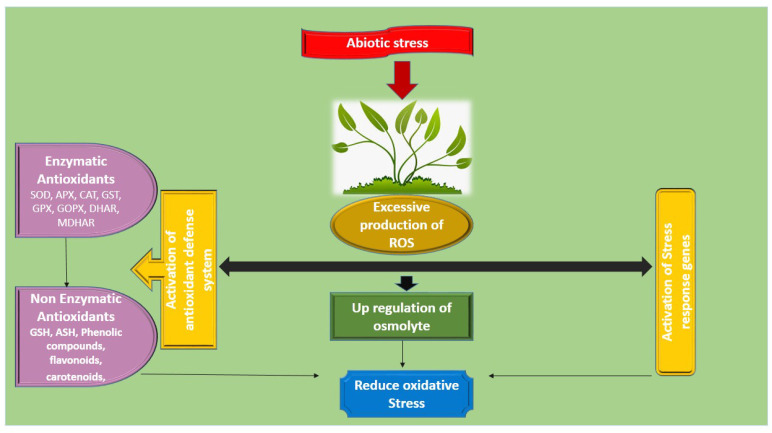
Abiotic stress response in plants.

Different metabolites are extensively present in plants, they regulate the growth of plants and support them to survive under environmental stress conditions [87]. These compatible osmolytes maintain plant growth and cell enlargement in stressful environments. Glycine betaine is one such organic osmolyte. Glycine betaine has significant importance in biological and morphological processes in plants. Accumulation of GB in plants increases the yields in terms of number of seeds, fruits and flower size [88]. A number of studies has shown that GB enhances growth of the plant by growing photosynthesis rate and chlorophyll levels in various plants like tomato [67], wheat [76], cotton [82] sweet potato [89] and soybean [90]. Utilization of GB considerably promote the plant growth via boosting up osmolyte accumulation and antioxidant potential [91]. Plants normally have active defense system (antioxidant system) which controls the oxidative stress [35], it includes both enzymatic and non-enzymatic system, and they actively take part in scavenging of ROS (Figure 3). The antioxidant system takes active part in promoting the growth of different plants species through controlling the excessive over-generation of ROS during both non-stressed and stressed environment [35,92,93,94].

In plants, GB plays a vibrant role in stimulation of the antioxidant system [95]. In plants, GB has the ability to extensively boost up the activities and exercise of various antioxidant enzymes such as SOD, CAT and POD [70]. Furthermore, it was perceived that GB assists in improving the chlorophyll content and photosynthesis rate in plants [82,96]. Different studies have reported constructive role of GB in plant growth and biomass under heavy metal stress [95,97,98,99]. Islam et al. [100] investigated that glycine betaine significantly improves cell growth of tobacco plant under Cd stress. Exogenous application of GB extensively improve plant growth, biomass and rate of survival [101,102]. Glycine betaine shields photosynthetic pigments and chlorophyll contents which ultimately results in improved plant growth [103]. Different studies have reported that GB demonstrated encouraging effects on improvement and growth of several plants in stressed conditions [70,83,85,98].

## 6. Promotive Role of Glycine Betaine in Mitigating Heavy Metal Stress in Plants

Heavy metals are considered very important pollutants in the environment and most of them are very toxic even at very low concentration [104]. A high concentration of HMs uptake in plants causes the excessive generation of ROS which may results in oxidative damage to cellular compartments of the plants [105,106]. Therefore the understanding antioxidant mechanism, oxidative stress along with its mechanism of alleviating the oxidative stress are crucial for plants under heavy metal stress. Glycine betaine also act as osmoprotectants inhibiting the production of ROS and free radicals [107]. Alteration of gene expression has been observed in different studies under HMs stress [55,108]. Glycine betaine regulated all the required gene expressions that create additional antioxidants enzymes SOD, POD and CAT and successfully scavenge the unwarranted ROS in perennial ryegrass under Cd stress [97]. Earlier studies have studied the positive impacts of GB on the mitigation of metals stress in various plants species [81,82,97,109]. Exogenous application of GB reduces the harmful effect of Cd toxicity on vital growth of the *Lemna gibba* L. plant [110]. Glycine betaine successfully enhances HMs stress tolerance in pants and keeps the cells of plants free from the heavy metal toxicity. There are different studies demonstrating the constructive role of GB application in mitigation of heavy metal stress in various plants (Table 1).

### 6.1. Improvement in Plant Growth and Biomass

When open to heavy metal toxicity, plants face growth retardation and inhibition. HMs stress results in the considerable reduction of plant growth, biomass, number of leaves, plant height, weight and length of root and stem [111,112]. Moreover, HMs inhibit the process of transpiration, replication and thus altering the growth and cell division of plants [27]. However, application of GB not only acts as an osmoprotection but also enhances plant growth when exposed to HM stress. Glycine betaine improves the plants growth under heavy metal stress by escalating chlorophyll contents and reducing the oxidative damage [47]. Glycine betaine safeguard the photosynthetic pigments and machinery that in results in improvement in growth of the plants [116,117,118]. Glycine betaine application improves the adverse effects of Cd stress in cotton seedlings, efficiently alleviating the Cd induced decline in plant biomass and its growth. Foliar application of GB helps plants to maintain the healthy growth and cell division [82].

Foliar application of GB effectively alleviates the chromium’s noxious effects on wheat and improved the plant growth, roots and shoots weights [81]. Foliar application of GB boosts the growth potency through enhancing root/shoot length, along with dry biomass of the plant [119]. Higher concentration of Cd reduces the both dry and fresh weight of the tobacco, whereas application of GB alleviates the Cd toxicity and improved plant biomass and growth under Cd stress [120]. Treatment of GB significantly mitigate the inhibitory adverse impacts of Cd on important crop wheat (*Triticum aestivum* L.) growth attributes, and plants showed better dry biomass of shoots under Cd stress [111]. Lead induce growth inhibition were reported in several plants such as Perennial ryegrass (*Lolium perenne* L.) [100] and harmal (*Peganum harmala* L.) [121]. Zouari et al. [109] described that supplementation of GB proved to be useful in preventing the Pb content in tissues of olive trees and thus decreasing its intimidating impacts on plant growth. Similarly application of GB restricted the Pb content in juvenile tissues of cotton plants and mitigated its inhibitory adverse effects on growth characteristics [98]. 

### 6.2. Enhancement in Rate of Photosynthesis

General heavy metal stress is known to change the plant water relation, which may possibly affect the rate of photosynthesis, ascent of sap, mineral nutrition, water uptake, functioning of stomata and slowdown of chlorophyll biosynthesis and eventually results in restriction of successful photosynthesis [122,123]. In plants, photosynthesis is highly susceptible to HMs stress [124]. Heavy metals toxicity increases the generation of ROS. Heavy metals interaction with different electron transport activities in the mitochondrial membrane and in cellular compartments of chloroplast results in production of ROS [125]. Different studies has reported that toxicity of HMs to various physiological process of plants occurs through (ROS)-prompted lipid peroxidation and also by its by products such as linolenic acid-13-ketotriene, 12-oxo-phytodienoic acid and acrolein which may vigorously distress rate of photosynthesis and PS II [103,126].

Glycine betaine is plentiful mostly in chloroplast where it protects the thylakoid membrane and sustains the photosynthetic activities [127]. Glycine betaine contributes to control of the cytoplasmic dehydration and uphold turgor pressure in leaves of plant subjugated to water deficient state, thus conserving the eminent photosynthetic activities [128]. Exogenous application of GB improves the growth attributes of sorghum by stimulating the leaf expansion, enhancing turgidity and increasing the creation of photosynthetic pigments [129]. The exogenous GB supplement mitigated the decline in photosynthetic activity under Pb stress in Cotton plant. GB boosts the Pb tolerance by enhancing the photosynthetic activities and synthesis of chlorophyll [99]. It is recognized that GB protects the plants photosynthetic activities by raising stomatal conductance, preserving the RuBisCo enzyme activity and conserving the ultrastructure of chloroplast against environmental stress [130].

### 6.3. Up-Regulation of Antioxidant Defense System

**Figure 4:**
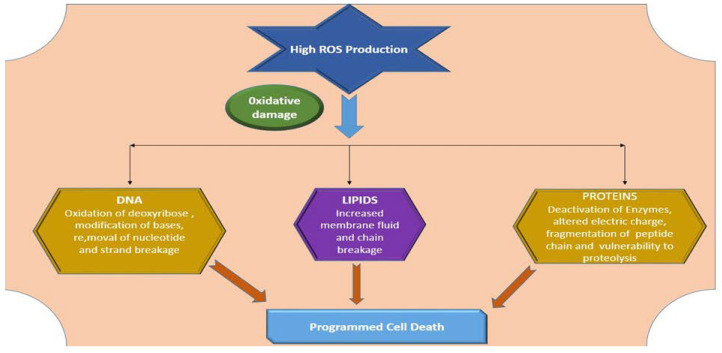
Reactive oxygen species (ROS)-induced oxidative damage to DNA, lipids and proteins.

Plants consecutively produce excessive and superfluous ROS due to HMs stress [131]. ROS are key constituents of the signaling pathways, which operate as main regulators of cell physiology and cellular response of plants as a results of several harsh environmental stresses [132]. In several plants, HMs ions attach to protein groups and are competent enough to replace defined cations in binding spots, resulting in the generation of ROS and inactivation of enzymes, that ultimately cause oxidative stresses such as oxidation of amino acids, degradation of proteins, inhibition of vital enzymes, damage to DNA and RNA and membrane lipids peroxidation [133] as shown in Figure 4. An abrupt rise of ROS at an intracellular level is caused owing to the unevenness between scavenging and production of ROS [134,135]. Therefore, generation of ROS should remain in compatible limits within plants.

Plants do have a definite scheme of antioxidants like CAT, POX, GSH and SOD functions as reducing agents which cease oxidation reaction by eliminating free radicals. Generation of such molecules inhibits the incidence of oxidative burst [136]. Different reports stated that GB has a task like an osmoslyte, and may increase the antioxidant system to minimize the adversative impacts of HMs toxicity in plants [83,109]. Exogenous application of GB in cotton enhanced the antioxidant system by reducing the oxidative stress, as proved by the declined generation of H_2_O_2_ contents, electrolyte leakage (EL) and MDA level in leaf and root of the plant grown under Cd stress conditions [82]. Lou et al. [96] stated that the activities of different operative antioxidant enzymes like SOD, CAT, POX were significantly improved when GB was applied exogenously to *Lolium perenne* L. under Cd stress. Similarly supplementation of GB promoted the antioxidant enzymes effective activities and restrained Cr accumulation and oxidative damage in wheat [81]. 

Plants also possess another shield mechanism to inhibit the destructive effects of ROS (i.e., generated as a result of HMs toxicity) is the prevalence of the ascorbate-glutathione (ASC-GSH) cycle [49]. Successful treatment of GB upregulate enzymes activities in the ascorbate-glutathione cycle. Activities of DHAR (dihydro ascorbate reductase), APX (ascorbate peroxidase), MDHAR (monohydro ascorbate reductase), glutathione S-transferase (GST), glyoxalase (Gly I,II) and glutathione peroxidase (GPX) were considerably improved by GB application in mung beans under Cd stress [96]. Increased in APX (ascorbate peroxidase) with the application of GB has been studied in cultured tobacco cells under Cd stress [115] and also reported in cotton under Pb stress [97].

### 6.4. Upgrading of Metal and Mineral Uptake

Glycine betaine mitigates the lethal impacts of HMs by improving the resistance mechanisms of plants under stressful conditions. Plants have several mechanisms and ways to regulate and sustain homeostasis of metal cellular uptake and much higher accumulation of liberated metal ions [81,137]. Supplementation of GB significantly reduces the concentration of Cr and its total uptake by mung beans in its parts as compared to the pertinent Cr treatment alone [83]. Decrease in Cr uptake in several parts of the plant body occurred due to the defensive function of GB for the plant cell membrane for that reasons, a smaller amount of Cr penetrates the cytoplasm [138]. Application of GB extensively reduces the concentration of Cd in the roots, stem and leaf of the cotton plant [82]. Moreover, the reduction in total Cr accumulation in plant along with the application of GB occurs due to the reasonable competition between Cr and other critical nutrients [139]. Similarly, a decline in several metals including Cd, Pb and Cr has previously been testified in various plant like wheat [81], mung bean [96], cotton [97] and rice [140] with applications of GB.

Foliar application of GB increases the concentration of important nutrients such as potassium and sodium ions in both the shoot and root of wheat (*Triticum aestivum* L.) [118]. Mahmood et al. [141] also reported an enhanced content of important ions like Na^+^ and K^+^ with higher application of GB in wheat plant. Decrease and increases in the concentrations of micronutrients accumulation by the plants relies upon the difference of GB application approaches and disparity among plant species [81]. The presence of these micronutrients ensures effective plant growth, plant metabolism, synthesis of chlorophyll, seed and fruit development and production of carbohydrates while their shortage encourages irregular growth in plants [142].

### 6.5. Alleviation of Electrolyte Leakage (EL), Malondialdehyde (MDA) and Hydrogen Peroxide (H_2_O_2_)

Malondialdehyde (MDA) is believed to be an indicator of the generation of free radical and cytotoxic outcome of lipid peroxidation in stressed cells [143,144]. MDA is renowned biomarker of the complex oxidative stress [145]. Heavy metal toxicity leads to increases in MDA contents in plants [146]. Antioxidant machinery performs a vigorous part in the decline of MDA contents under heavy metal stress [147,148,149]. Increases in MDA content, H_2_O_2_ and EL will breakdown the homeostatic ROS balance which will eventually decrease the actions of vibrant antioxidant enzymes under heavy metal stress. Similarly, previous studies also reported the same effects on antioxidant enzymes and production of ROS in various plants [150,151,152]. Nevertheless, application of GB improves the antioxidant enzymes activities and headed to diminish the oxidative damage as confirmed through declined generation of hydrogen peroxide, EL and robust MDA level in both roots and shoots of the plants under metal stress [81,151].

Glycine betaine encourage the decline of H_2_O_2_, EL and MDA levels which specifies that GB effectively mitigates the detrimental impacts of Cd toxicity in cotton [82]. Supplementation of GB (10 mM) significantly reduces the MDA content induced by the Cd toxicity in cultured tobacco cells [118]. Oxidative stress induced by Pb on olive trees was considerably mitigated via exogenous application of GB through considerable reduction in EL, lipid peroxidation (TBARS) and hydrogen peroxide (H_2_O_2_) in all tissues analyzed [109]. Similarly, exogenously applied GB effectively detoxifies hydrogen peroxide (H_2_O_2_) by increasing antioxidant enzymes activities in *Phanerochaete chrysosporium* L. under Cd stress [153]. Application of GB reduces the malondialdehyde, hydrogen peroxide and EL conferred resistance in opposition to heavy metal stress by promoting preemptive activities of all important antioxidant enzymes.

## 7. Involvement of Glycine Betaine against Combinations of Abiotic Stresses in Plants

Plant species naturally accumulate GB as their main osmolyte once they are subjected to various type of abiotic stresses. Glycine betaine accumulate in many species of plants such as wheat (*Triticum aestivum* L.) [81], cherry tomato (*Solanum lycopersicum* L. *var. cerasiforme*) [154], sugar beet (*Beta vulgaris* L.) [155], maize (*Zea mays* L.) [156] and tomato (*Solanum lycopersicum* L.) [157]. Exogenously application of GB has been extensively examined to improve its capacity to alleviate the abiotic stress condition including drought, salt, and temperature. Induction of chilling stress tolerance has also been previously reported in sugarcane, loquat fruit, sweet pepper with the application of GB [158,159,160]. Application of GB reduces the chilling stress in potato plants by improving ROS scavenging, reducing chlorophyll bleaching and stabilization of photosynthetic activities [161]. Application of GB improves the performance of photosynthetic characteristics’ of bread wheat under drought stress [78]. Different studies have reported that GB improved tolerance against drought stress due to its active participation in osmotic adjustment in different plants [162]. Under severe drought stress, GB helps to uphold osmotic adjustment, sustain the sustainability of membranes and boost defense system in bentgrass [80].

Salt stress is also a big challenge for prosperous agriculture. Crops yields frequently decreased because crops fall short to survive salinity [163]. Glycine betaine also enhances salt tolerance in different plants. Its application successfully reduces salt stress in plants like tomato [164], onion [165], rapeseed mustard [166]. The number of nodules, activity of nodules and biological process of nitrogen fixation considerably reduce under salt stress. Under salt stress, GB application considerably enhances the particular nodule activity and nitrogen fixation [167]. Utilization of GB increases plant height, biomass, root length and total chlorophyll in rice plant under harsh salt stress [168]. The application of GB further enhances proline content, soluble sugar along with successful improvement in vital antioxidant systems which offer better conditions for the growth of plants exposed to salinity stress [169]. Plants treated with GB also retain greater antioxidant enzymes movements that restrict oxidative stress under salinity [165,170].

Compatible solutes including polyamines, proline, GB, and soluble sugar accumulation in plants are positively correlated with low temperature stress tolerance [171,172] and extreme temperature [64,173] in plants. Glycine betaine enhanced the growth of potato [174], maize [175], chickpea (*Cicer arietinum* L.) [176], and cotton [177] under chilling stress conditions. Cooling (<10 ℃) is particularly harmful to the development of chickpea, especially during the reproductive stage. Chilling causes a restriction in pollen tube growth, abnormal functions in male and female gametes and flower abortion. Chilling stress damage to membranes, a decrease in cellular respiration, increase in ROS, the elevation of abscisic acid and cryoprotectants such as GB safeguard the proactive activities of several enzymes and vital proteins, helping in stabilization of membranes structure and maintaining the photosynthetic apparatus under freezing temperatures during chilling stress. As reported previously, accumulation and uptake of GB is linked with the cold tolerance in several species of plants [178].

Foliar application of GB during the bud stage of chickpea showed much better improvement in flowering in the form of successful pollen germination, active pollen viability, tremendous growth in pollen tube, greater receptivity in stigma and extensive viability of ovule in chilling stress conditions. On the other hand, during pod filling, GB treatment resulted in an increase in the number of seeds and its yield, seed weight and pods were significantly improved after treatment. Glycine betaine greatly induced cold stress tolerance in plants through effective improvement in overall leaf water, sucrose and chlorophyll content, meanwhile, it also reduced the ROS and contents of abscisic acid as well [177]. Some plant species such as barley (*Hordeum vulgare* L.) [179] and spinach [180] synthesize more GB in different compartments of chloroplasts than plants like rockcress (*Arabidopsis*) [181] and tobacco [182]. Exogenously GB application to chickpea plant, increasing the flower behavior, pod formation and many yield parameters, provides a partially cold tolerance in the plants [176].

Cotton crops (*Gossypium hirsutum* L.) are very prone to chilling stress [183]. Thus, chilling is considered a major stress that may intimidate the overall production of cotton. It is important to increase the chilling tolerance of cotton production during these periods. Seed germination was significantly improved by glycine betaine application under chilling stress. Seed priming with GB enhanced stress tolerance in plants [184,185]. Seed priming with GB make the cotton seedlings more resilient to chilling stress. Seedlings obtained from GB-treated seeds show higher SOD, CAT and APX activities, thus lowering the H_2_O_2_ content of the leaves and reducing cell damage. Treatment with GB modulates the manifestation of some important genes that produce ROS-scavenging enzymes. While plants are in stress conditions, faster buildup of GB occurs in several plants and the content of GB is positively associated with increased tolerance. Application of GB to cotton seeds provides tolerance during the germination stage and vital four-leaf stage of seedlings [177].

## 8. Application Methods of Glycine Betaine

Different methodological approaches are used to apply the GB at a range of different concentrations, and it performs a substantial role in fortification and safeguarding of physio-chemical attributes of the plants under HM stress. Different types of GB application methods increase the yield, fertility and growth of the plants under stress and non-stress conditions [64,95]. There are different methods of GB application which can be directly used as plant growth supporters.

### 8.1. Foliar Application of GB

Foliar application of GB is suggested as a valuable mean of inducing tolerance in plants under stress conditions with destitute solute accumulation [118]. Different factors define the usefulness of foliar treatment of GB such as concentration of GB applied, application period and types of plant species on which GB is applied [64]. Foliar supplementation of GB substantially alleviate the harmful results of Cr toxicity on mung bean and enhanced biomass, growth along with total chlorophyll contents [83]. A similar improvement in biomass, chlorophyll content and uptake of essential nutrients were also examined under foliar exogenously applied GB under Cd stress in amaranth [86]. Foliar application of GB also improve the physiochemical attributes under Cd stress by reducing the H_2_O_2_, level of MDA and superoxide radicals (O^2−^) in two cultivar of wheat [110]. Foliar treatment of GB improve the antioxidant enzymes’ activities and reduce the oxidative stress induce by the Cr stress in wheat plant [81].

### 8.2. Pre-Sowing Seed Treatments 

Different studies support the fact that the application of GB is equally effective at very early growing phases of seed germination and seedling establishment [186,187]. Survival of seedlings and germination of seeds under various kinds of environmental stress are vital for crop yield and its establishment [188], which can be efficiently improved by treating the seeds with the application of GB [189,190]. Seed treatment with the supplementation of GB enhanced the activity of the PSII center. Glycine betaine application mitigates the prohibitory impacts of environmental stress during the reparation of PSII center by speeding up the establishment of D1 protein [191].

Glycine betaine seed treatment protects the chloroplast membrane, increases the osmotic adjustment, preserves the photosynthetic pigments and also sustains the turgor pressure by accumulation of both inorganic osmolytes (Mg, Ca and K ions) and organic osmolytes (total amino nitrogen, proline, total soluble proteins and total soluble sugars) [192]. The water potential of plant tissues and cells is harmfully affected under different kind of osmotic stresses. Korkmaz et al. [193] stated that pre-sowing seed treatment with 5mM of GB considerably enhanced the relative water content (RWC) and leaf water potential of pepper seedlings under salt stress. Arafa et al. [194] also described that pre-soaking of sorghum grains with GB improved the germination rate in contrast with the control treatment. Seed pre-sowing treatment along with GB significantly motivated the germination of seeds and improvements in antioxidant systems of the plants. Application of GB at seeds level sustained a higher level of antioxidant enzymes including SOD, APX and CAT activities and it retain H_2_O_2_ contents at lower levels in leaves, thus preventing the level of injury occurred to cell membrane in cotton seedling [177].

## 9. Role of Glycine Betaine in Crop Improvement 

The methods of orthodox plant breeding have altered the use of physiological assortment parameters and rely on genetic variability that is already present [195]. The significant variation in the GB contents of wheat [196], barley [197] and maize [198] verified that the behavior of GB in plants was a quantitative character. It was emphasized that there was considerable genetic diversity for GB in several heterotic groups of maize and a recessive allele of a single nuclear gene was responsible. Betaine aldehyde dehydrogenase (BADH) and choline oxidase (COD) are key enzymes which have been used to converse glycinebetaine synthesis in plants, which normally does not synthesis glycinebetaine. Genetically modified tomato plants with choline oxidase and BADH enzymes showed much better development in size and flowering [199]. Non-significant maternal or cytoplasmic effects were determined in the quantitative genetic studies for GB. The higher additive gene effect than non-additives was estimated by incomplete diallele analysis with eight low- and five high-betaine parents in barley. Similarly, the results of scaling tests (generation mean analysis of the parental, F1, F2, and backcross generations) showed a predominantly additive trait and moderately narrow sense heritability degree [195,197]. It is advisable to select individual plants for GB in early generations (F_2_–F_3_).

## 10. Genetic Engineering 

Genetic manipulation is one of the most important techniques used now days for the creation of crop resistance to different environmental stresses. Different crops have been established for the upgrading against stress confrontation via increasing generation of different hormone, antioxidant enzymes and organic osmolytes [200,201]. Glycine betaine is an important osmolyte that defends the plant against the detrimental effects of environmental stresses. Genetic engineering creates transgenic plants which contains various genes for the GB biosynthesis pathway, which ultimately develops increased resistance to a widespread environmental strains through various phases of plant development [202]. Choline oxidase (COD) and betaine aldehyde dehydrogenase (BADH) are two main enzymes whichh are chiefly used to create GB synthesis in that plant which does not normally synthesise GB. Zhang et al. [203] described that genetically transformed tomato plant with codA, BADH transgenes showed much better rate of photosynthesis, better chlorophyll content along with higher assimilates content as compared to non-transformed wild plant. 

During genetic manipulation gene products primarily target the chloroplast in *cod*A transgenic plants. Transgenic plants predominately accumulate GB in chloroplast, and display tolerance to different types of abiotic stresses [67]. Transgenic reports over the years have demonstrated that those plants which generate additional GB have improved the resistance to various environmental stresses [204,205]. The important gene (*BADH*) betaine aldehyde dehydrogenase plays a very crucial function in plants under stressful conditions. Qin et al. [206] stated that BADH transgenic soybeans have shown a 6–17% increase in germination index, POX activity enhanced by 1–7% and decreased in contents of MDA by 1.5–13% in comparison to the control treatment. Increase in the accumulation of GB through genetic engineering could be effectively exploited as an essential implement to mitigate the heavy metal stress confrontation in plants. Cloning of (BADH) genes will further upon up the new horizons to create stress-tolerant crop cultivars [207].

## 11. Conclusions

Glycine betaine is a key organic osmolyte, which usually accumulates in a diverse range of plants against different environmental stresses. Also, it assists the plants to recover from severe stress quickly. In this review, we aimed to explore the impacts of GB on growth traits along with the mechanism engaged in plants to mitigate the metals stress. Exogenous application of GB boosts and improves yield and growth well as physiological attributes of plants. Glycine betaine application reduces heavy metals toxicity by increasing the robust activities of imperative antioxidant enzymes. Exogenously applied GB lessens the ROS formed in various plants under heavy metal stress. Thinking about the ability of exogenous GB as scavenger of ROS, it providentially transforms into expressive means to oppose the unfavorable impacts of environmental stress, thus reducing annual loss of agriculture. Furthermore, GB application maintains the water relation and turgor pressure in plant cells and also raises the rate of photosynthesis. The foliar method of GB application was found to be more efficient in alleviating the heavy metal stress and improving the growth attributes of the plants. Application of GB showed ameliorative impacts on growth characteristics of plants. Further investigation is required to fill the gap concerning the physiological response of GB and its application during signal transduction paths in several plants exposed to heavy metal stress. A wide range of experimental studies are required to examine how GB improves the nutrient uptake at molecular level and how GB collaborate with physio-chemical processes of the plants. Genetic manipulation and further understanding of inducible GB genes and their products could easily improve our knowledge of GB improvement to stress tolerance and resistance in plants.

## Figures and Tables

**Table 1:** Summary of the application of glycine betaine on various plant species under heavy metal stress.

Plant Species	Heavy Metal Stress	Effect of Exogenous Glycine Betaine (GB)	References
Wheat	Cr Stress	Glycine Betaine (GB) improved chlorophyll contents, biomass, growth characteristics and protein content.	[81]
Cotton	Cd Stress	Glycine Betaine (GB) boosted the plant growth, improved activities of antioxidant enzyme and rate of photosynthesis	[82]
Mung Bean	Cr Stress	Glycine Betaine (GB) improved plant growth.	[83]
Amaranth	Cd Stress	Glycine Betaine (GB) significantly encouraged the rate of photosynthesis in edible amaranth and considerably improved the chlorophyll content of leaves.	[89]
Perennial Ryegrass	Cd Stress	Glycine Betaine (GB) improved stability of cell membrane via decreasing lipid membrane oxidation.	[97]
Cotton	Pb Stress	Effectively improved the gas attributes and plant growth under Pb stress.	[99]
Wheat	Cd Stress	Glycine Betaine (GB) improved fresh biomass of roots and shoots.	[111]
Cucumber	Al Stress	Glycine Betaine (GB) showed significant protective effect on chlorophyll content.	[112]
Asian Rice	As Stress	Glycine Betaine (GB) increased the GST and GRX gene expression alongside As stress.	[113]
Sorghum	Cr Stress	Glycine Betaine (GB) improved the quality and total yield of sorghum.	[114]
Tobacco	Cd Stress	Glycine Betaine (GB) reduces the stomatal closure, accumulation of malondialdehyde (MDA) and damage to leaf.	[115]
